# Pup mortality in New Zealand sea lions (*Phocarctos hookeri*) at Enderby Island, Auckland Islands, 2013-18

**DOI:** 10.1371/journal.pone.0225461

**Published:** 2019-11-21

**Authors:** Sarah A. Michael, David T. S. Hayman, Rachael Gray, Ji Zhang, Lynn Rogers, Wendi D. Roe

**Affiliations:** 1 Sydney School of Veterinary Science, The University of Sydney, Camperdown, New South Wales, Australia; 2 School of Veterinary Science, Massey University, Palmerston North, New Zealand; 3 Molecular Epidemiology and Public Health Laboratory, Hopkirk Research Institute, Massey University, Palmerston North, New Zealand; Animal Health Centre, CANADA

## Abstract

New Zealand sea lions (*Phocarctos hookeri*) are an endemic and endangered species. Pup mortality at Enderby Island (50.5°S, 166.28°E) in the New Zealand sub-Antarctic has been well studied, with subsequent investigations yielding more intricate detail of the causes of mortality, as new diagnostic methods become available. *Klebsiella pneumoniae* was first reported in 2001–02 at this site, causing a pup mortality epizootic and is now known to be present at several colonies. This bacterium is a common mucosal commensal of humans and animals, however the agent found in pups at necropsy is a hypervirulent strain, readily recognised in microbial culture as being hypermucoviscous. Infection causes septicaemia with a common syndrome of subsequent meningitis and polyarthritis. This investigation uses histopathology and microbiology, with new modalities such as matrix assisted laser desorption/ionisation—time of flight mass spectrometry to show that *Klebsiella* septicaemia could have historically been, and continues to be, the most important cause of pup mortality, but has been previously underrepresented due to the often cryptic presentation and sometimes peracute course of disease. Hypermucoviscous *K*. *pneumoniae* should be considered a serious threat to pup survival in the species, causing on average 60.2% of pup deaths annually at Enderby Island between 2013 and 2018, with likely more continuing mortality following pup dispersal and the cessation of the summer monitoring season. Less common causes of death included starvation (14.8%), trauma/asphyxiation (9.9%) and other infections (7%). This study forms the basis for further evaluation of risk factors for pup mortality in the species, with a view to developing active mitigation.

## Introduction

**Fig 1:**
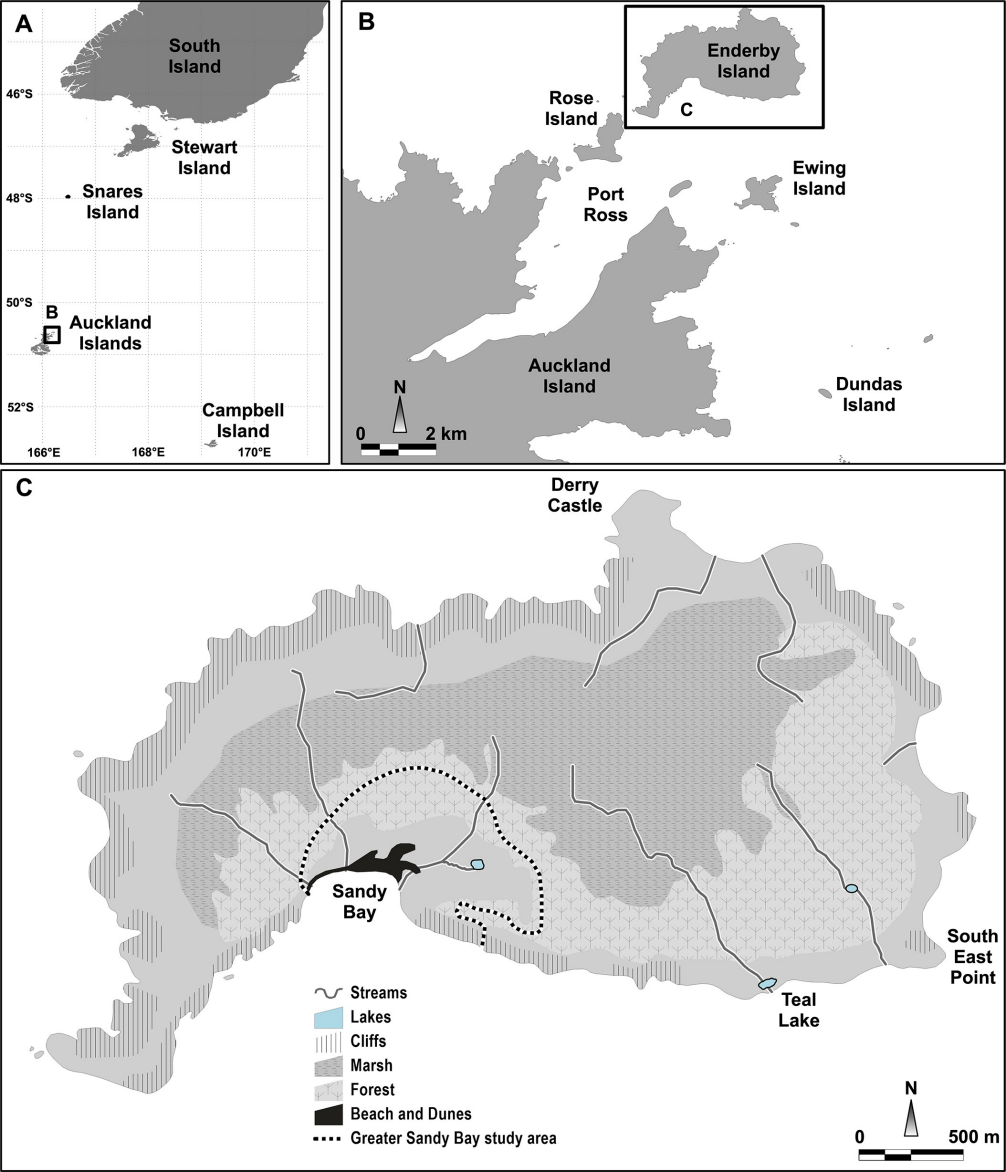
Map of New Zealand sea lion (*Phocarctos hookeri*) breeding areas. (A) Outline of all breeding sites, including the South Island of mainland New Zealand, Stewart Island, Auckland Islands and Campbell Island. (B) Breeding sites at the northern Auckland Islands including Enderby Island and Dundas Island. Port Ross islands are common dispersal sites. (C) Localities of Enderby Island including the study site, the greater Sandy Bay area.

The New Zealand (NZ) sea lion (*Phocarctos hookeri*), the only pinniped endemic to NZ, is rare and highly localised, with almost all breeding occurring in the outlying sub-Antarctic islands between 50-53°S. The Auckland Islands comprise the largest breeding site, with three colony locations including Enderby, Dundas and Figure of Eight Islands, with a further two significant breeding locations on the more remote Campbell Island (Fig 1). Sandy Bay, Enderby Island (50.5°S, 166.28°E; Fig 1C) is the best studied colony and while not the largest breeding location for the species, comprehensive research into demographics, disease and foraging, and a long term individual identification program (flipper tagging and intermittent passive integrated transponder tagging) have been conducted there since the 1994–95 field season. At Sandy Bay, pregnant females give birth to a single pup with the median pupping date of approximately 26 December and the population is highly synchronised, with 69% of pups being born within a two week period [1]. Pups are thought to be weaned around ten months of age [2], however some nurse for over a year (pers. obs). Pup production has declined since 1995, however, for the seasons covered here pup production has been stable, albeit at just over 50% of the apparent Auckland Islands population peak in 2000–01 [3].

Pup mortality at Enderby Island has been well studied [4–7], with increasing detail of the causes of death reported with the advent of new diagnostic methods. The emerging primary cause of pup mortality, *Klebsiella pneumoniae* was first reported in 2001–02 following an epizootic. Prior to this, the same agent has not retrospectively been isolated from archived necropsy samples ([6], unpublished data). This Gram negative, rod-shaped bacterium is a common mucosal commensal of animals including humans, however the agent found in NZ sea lion pups at necropsy is a hypervirulent strain, readily recognised in microbial culture as being “sticky” or hypermucoviscous (HMV). Since 2001–02, HMV *K*. *pneumoniae* infection has been recognised as a common cause of pup death at Sandy Bay [5, 6], but further analysis has shown that the true proportion of HMV *K*. *pneumoniae* deaths could have been, and continues to be, the most important cause of pup mortality, and is likely to have been underrepresented due to the often cryptic presentation and sometimes peracute course of disease [7]. Definitive diagnosis is possible with a combination of, and correlation between clinical, histopathological and microbiological analysis; all of which have been undertaken in this study.

Hypermucoviscous *K*. *pneumoniae* infection in humans is an emerging life-threatening community-acquired invasive disease, causing a hallmark syndrome of liver abscessation in the absence of biliary disease, as well as other syndromes including endophthalmitis, meningitis and necrotising fasciitis (reviewed by [8]). Early reports of human disease have centred in Asia, however an increasing number of cases are being reported in developed countries [9, 10] and in wildlife species around the world. Infection in California sea lion (*Zalophus californianus*) juveniles and adults causes pleuritis and suppurative bronchopneumonia [11, 12], while in African green monkeys (*Chlorocebus aethiops*), multi-systemic abscesses are the principal lesions [13].

The emergence of infectious diseases such as HMV *K*. *pneumoniae* at the Auckland Islands stronghold of NZ sea lion breeding highlights the importance of monitoring and thorough investigation of pup disease and mortality. This retrospective analysis aims to form the basis of current knowledge on recent trends in pup mortality in NZ sea lions at Enderby Island, with a view to identifying the most important targets for active mitigation and conservation management for this endangered species.

## Materials and methods

Collection and necropsy of NZ sea lion pups found dead at Sandy Bay, Enderby Island was undertaken yearly during the austral summers between 2013–14 and 2017–18. The greater Sandy Bay area (Fig 1C) was searched at least daily for the duration of each field season, which ranged from 45 to 92 days in length. Other sites around Enderby Island were also searched opportunistically. Researchers moved through the Sandy Bay colony between one and eight times daily to record resights of individual animals and identify pups with abnormal physical findings or unusual behaviour. When dead pups were located, they were transported back to the Sandy Bay base and necropsied as soon as was practicable (usually within 24 hours). Each pup was identified by sex and flipper tag or microchip if present and morphometric measurements were collected (weight, standard length, axillary girth, sternal blubber depth as described in [14]), before a full necropsy was undertaken by a veterinarian as described by Roe and colleagues [7]. In addition to standard necropsy procedure, hookworm (*Uncinaria* spp.) presence was determined by visually evaluating serially longitudinally opened sections of small intestine for the presence of adult worms. Haemorrhagic contents were subjectively graded (none, mild, moderate, severe) and presence or absence of serosal petechial or ecchymotic haemorrhage was noted. Representative tissues (lung, heart, liver, spleen, kidney, ovary or testis, stomach, small and large intestine, pancreas, adrenal gland, lymph node, tongue, skeletal muscle, diaphragm, trachea, cerebrum, cerebellum and cervical spinal cord) and samples of lesions were collected into a 10% formalin-seawater solution for later histopathological analysis. A selection of tissues (liver, lymph node, spleen, brain, lung and lesions) were also frozen in liquid nitrogen and subsequently stored at -80°C, until microbiological analyses.

Following transport to Massey University, Palmerston North, New Zealand, formalin-fixed tissues from all cases were trimmed, embedded in paraffin, sectioned at 4μm and stained with haematoxylin and eosin (H&E). Special stains were used when required, including Gram’s and Giemsa for further investigation of potential infectious agents. Representative frozen tissues from all cases were thawed and routinely cultured aerobically and in 5% CO_2_ at 37°C for 24 hours on MacConkeys and horse blood agars (Fort Richard Laboratories, Auckland, New Zealand). Pure and predominant growths were identified to species level with matrix-assisted desorption/ionisation–time of flight mass spectrometry (MALDI-TOF MS) from an ethanolic suspension. This was accomplished by homogenising approximately 2mm^3^ of bacterial colony from a pure overnight culture in 300μL sterile water, then mixing with 900μL high grade 100% ethanol. Targets were prepared according to manufacturer’s recommendations and spectra were measured using the Bruker Biotyper and the Bruker isolate database version 7 (7311 RUO). HMV *K*. *pneumoniae* isolates were defined as those confirmed as *K*. *pneumoniae* on MALDI-TOF MS (log score estimate of certainty >2.00), with a positive string test (viscous string >5mm, [15]). For further species confirmation of *Streptococcus* spp. by biochemical characteristics; mannitol, ribose and sucrose fermentation; bacitracin sensitivity; arginine dihydrolase and CAMP test were conducted in parallel. *Salmonella* isolates were serotyped at the New Zealand Enteric Reference Laboratory, Wallaceville, New Zealand. For isolates that were unable to be identified by the MALDI-TOF database, genomic DNA was extracted from a single colony using the QIAamp DNA MiniKit (Qiagen, Hilden, Germany). DNA was sequenced at Novogene (Beijing, China) using Illumina HiSeq^™^ X sequencing (2x150 base PE) after library preparation using the Nextera XT library kit (Illumina, San Diego, CA, USA). Sequences underwent Kraken2 [16] and 16S rDNA analyses to determine most likely species.

Definitive cause of death was determined using a combination of clinical history from colony observations, gross necropsy findings, histopathology and microbiology. Causes of mortality were categorised into the following groups: infection (HMV *K*. *pneumoniae* or other), starvation, trauma/asphyxiation, open, congenital and stillbirth/periparturient death. The open category included those cases where a diagnosis could not be made due to absence of pathological findings, extensive scavenging or decomposition of the carcass. In several cases more than one definitive cause of death applied and were designated a compound diagnosis. Infection cases were diagnosed based on gross and histological evidence of inflammation and infection in addition to a pure or predominant growth on culture from affected tissues. Drowning was diagnosed if the carcass was found submerged in a water body with gross necropsy evidence of significant aspiration of water and foreign material in small airways. Starvation cases were diagnosed if blubber depth was less than 3mm in combination with clinical history and absence of other significant lesions.

An anthelmintic treatment trial was run concurrently throughout the 2016–17 and 2017–18 seasons. Ivermectin (Ivomec^®^ Injection for Cattle, Sheep and Pigs, Boehringer Ingelheim Animal Health, Manukau City, New Zealand) was administered subcutaneously to 163 and 180 randomly selected pups born at Enderby Island during these seasons respectively, at a dose of 0.2mg/kg at approximately one week of age. The results of this study will be reported separately but should be considered for interpretation of pup mortality in these field seasons.

The chi-squared (χ^2^) test was used to determine associations between categorical variables, with *p* <0.05 the threshold for statistical significance. Ninety-five percent confidence intervals (95% CI) were calculated using the binom.test function for percentages and pois.exact for counts or rates in R (Version 3.5.2) [17, 18].

Procedures were permitted by the Department of Conservation, New Zealand (necropsies 39239-MAR, all other procedures were permitted as species management). All methods were approved by the Massey University Animal Ethics Committee (approval number 16/89) and Department of Conservation Animal Ethics Committee (approval numbers 249, 265, 276 and 304).

## Results

### General findings

Over five seasons, 316 dead pups were identified, of which 284 (90%) underwent necropsy. Those that were not necropsied were either too decomposed to facilitate an informative investigation or were unable to be retrieved from dense harems without putting live pups at risk of trampling. All dead pups were found in the greater Sandy Bay region, except for five (of which three were necropsied) found at Teal Lake, located approximately 2km east of Sandy Bay on the southern coast (Fig 1C). All dead pups had been born at Sandy Bay, except for 37 confirmed (on flipper tag) or presumed (found untagged after all Sandy Bay pups had been tagged) to originate from nearby Dundas Island (Fig 1B), from where mothers and pups begin to disperse in mid-January.

**Fig 2:**
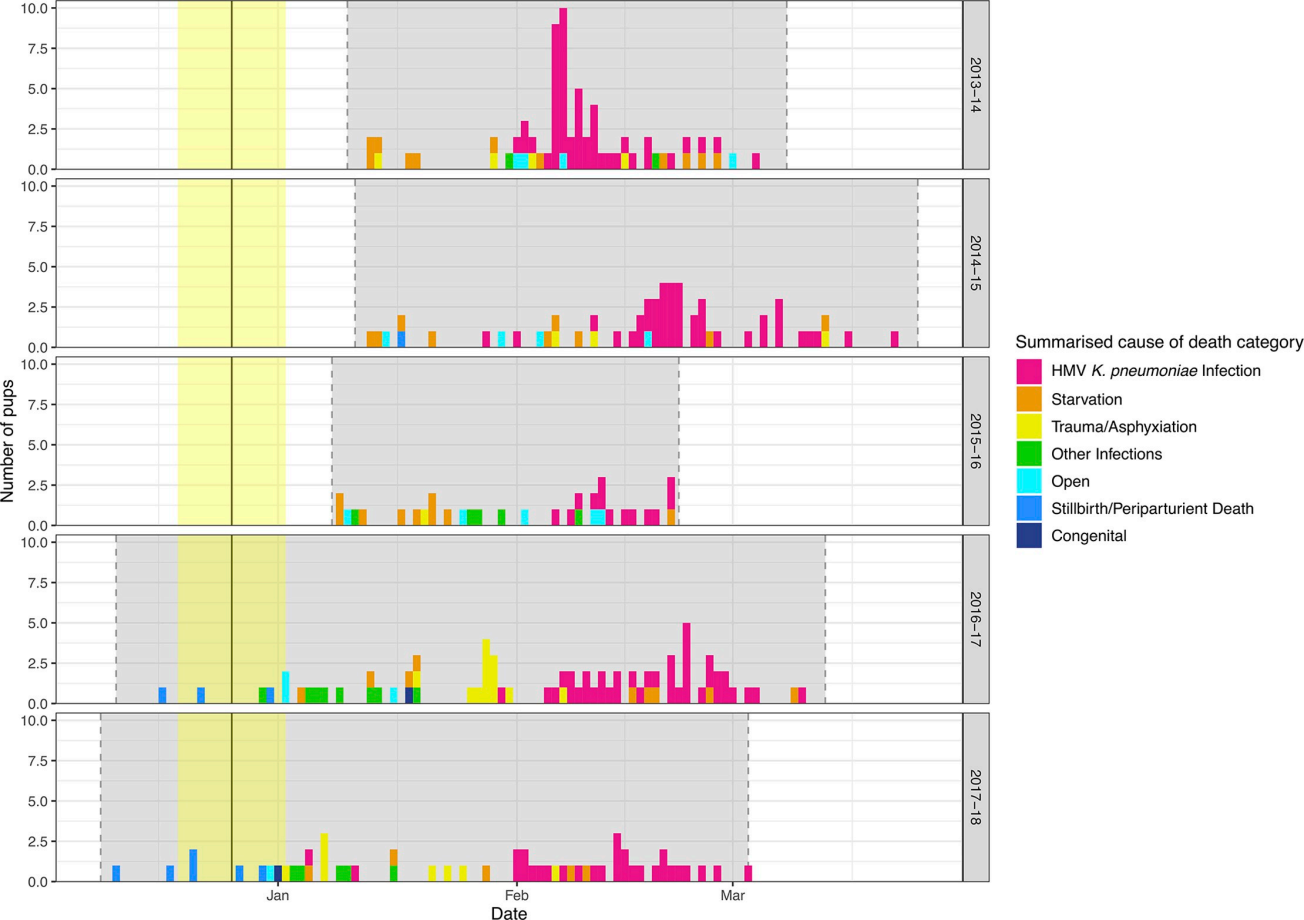
Histogram of summarised cause of death in New Zealand sea lion (*Phocarctos hookeri*) pups at Enderby Island presented by field season. Grey shaded area indicates the monitored period of each field season and yellow shaded area indicates the approximate timing of 69% of pupping with median pupping date of 26 December [1].

**Fig 3:**
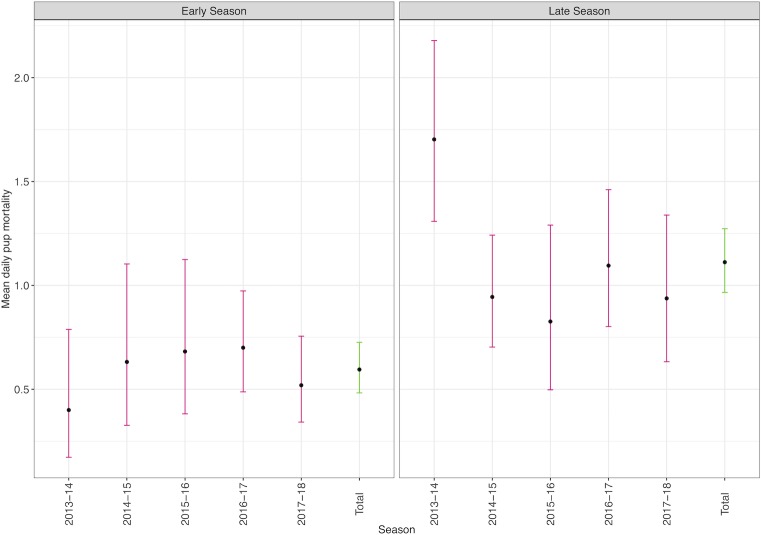
Mean annual daily mortality rate and 95% confidence intervals for New Zealand sea lion (*Phocarctos hookeri*) pups at Enderby Island for early and late season periods when the observed field season is divided at 30 January.

Pup mortality varied over the observed period as summarised in Table 1. The length of each field season also varied, with the 2013–14, 2014–15 and 2015–16 seasons being shorter, beginning in early January after the majority of pups had been born (Fig 2). The 2015–16 season also ended earlier compared to the other years, accounting for less than half the length of the longest season such that total mortality was likely underestimated, as early and late season mean daily mortality rates were similar to the other years (Fig 3; early/late season division at 30 January). Over all years, early season (0.6, 0.48–0.76 95% CI) and late season (1.11, 0.97–1.27 95% CI) mean daily mortality rates were significantly different. Despite the variation, the total mortality in 2013–14 and 2014–15, as well as 2016–17 exceeded the second of the two historic “epizootic” years (2002–03; 21.2% at end of February). In both years, over 69% of pup mortality was caused by HMV *K*. *pneumoniae* infection (Table 2). In 2013–14, 19 pups were found dead in a two-day period in a ‘spike’ of HMV *K*. *pneumoniae* mortality (Fig 2). Indeed, the 2013–14 late season was the only part of any season in this study to have a significantly different late season daily mortality rate to any other (Fig 3). The inclusion or exclusion of this data point did not change the outcome that the total late season daily mortality rate was significantly higher than the early season.

**Table 1:** Summary of New Zealand sea lion (*Phocarctos hookeri*) pup births and deaths at Sandy Bay, Enderby Island from 2013–14 to 2017–18. Total pups born was determined by a direct count [3].

Season	Total pups born	Total pups dead	Length of field season (d)	Total mortality (%)	Total pups necropsied	Sex ratio of necropsied pups (M:F:U)^#^
2013–14	290	71	57*	24.5	69	41:27:1
2014–15	286	63	73*	22.0	58	32:27
2015–16	321	34	45*	10.6	33	19:12:2
2016–17	349	81	92	23.2^~^	75	32:43
2017–18	332	57	84	17.2^~^	49	24:25

*Field seasons that began in early to mid-January, where researchers were not present throughout the pupping period.

^#^Sex ratio of necropsied pups is presented as male: female: unknown.

^~^Intervention was undertaken by treating approximately 50% of pups with ivermectin which could have influenced mortality.

**Table 2:** Summary of annual proportions of HMV *K*. *pneumoniae* deaths in New Zealand sea lion (*Phocarctos hookeri*) pups necropsied at Sandy Bay, Enderby Island for field seasons between 2013 and 2018.

Season	Total necropsies [untreated]	HMV *K*. *pneumoniae* cases [untreated]	HMV *K*. *pneumoniae* % [untreated %]	Date of first HMV *K*. *pneumoniae* case	HMV *K*. *pneumoniae* male cases (95% CI)	HMV *K*. *pneumoniae* female cases (95% CI)
2013–14	69	48	70%	1 February	66% (51–79%)	34% (21–49%)
2014–15	58	41	71%	28 January	59% (42–74%)	42% (26–58%)
2015–16	33	13	39%	6 February	62% (32–86%)	39% (14–68%)
2016–17	75 [54]	40 [30]	53% [56%]	31 January	40% (25–57%)	60% (43–75%)
2017–18	49 [33]	29 [22]	59% [67%]	1 February*	35% (18–54%)	66% (46–82%)
Total	284 [247]	171 [154]	60% [62%]		52% (45–60%)	48% (40–55%)

Square brackets indicate totals excluding pups that were treated with ivermectin.

*Outliers on 5 January (coinfection) and 11 January

### HMV *K*. *pneumoniae* infection

**Fig 4:**
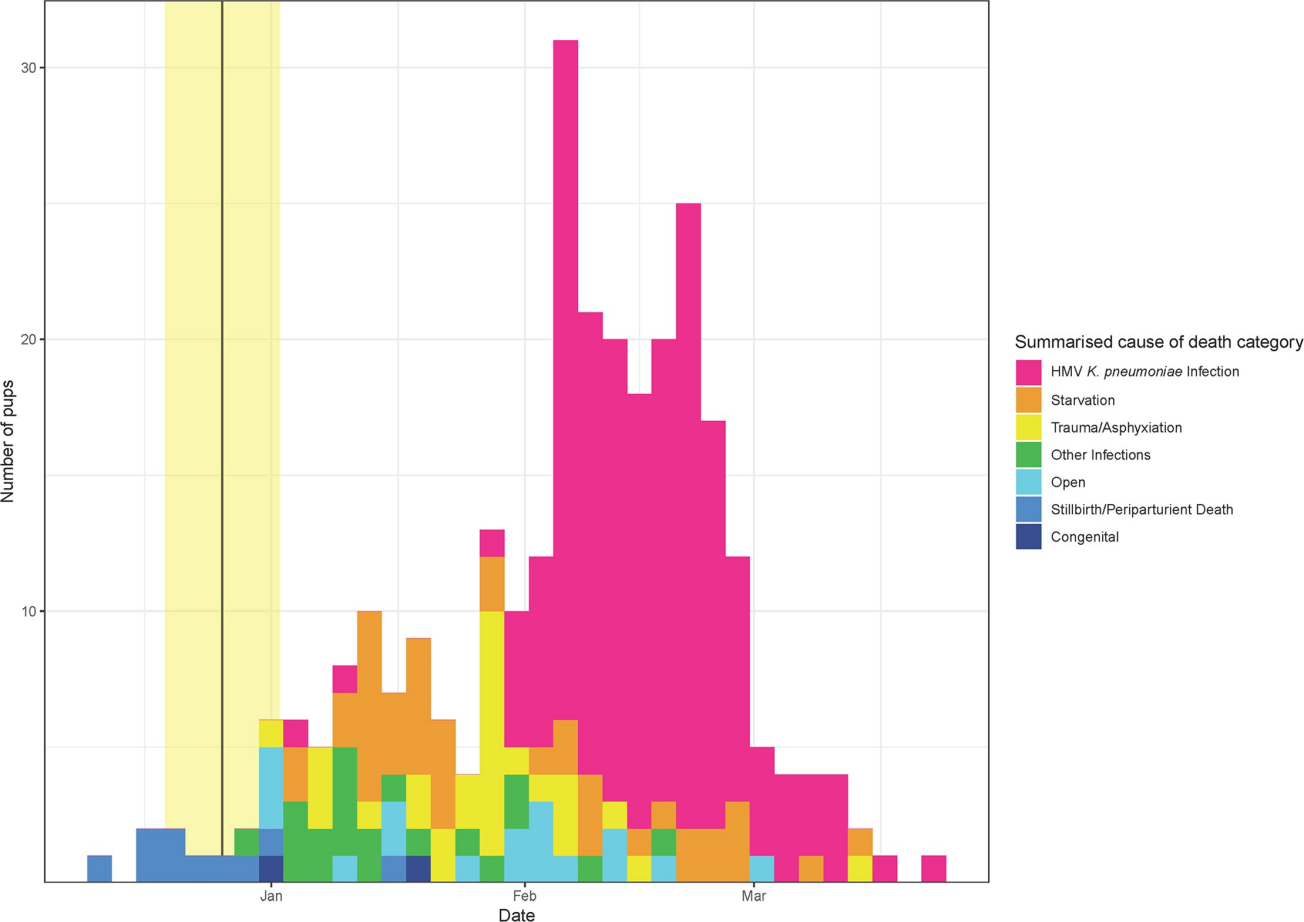
Histogram of summarised cause of death in New Zealand sea lion (*Phocarctos hookeri*) pups at Enderby Island with seasons 2013–14 to 2017–18 combined. The yellow shaded area indicates the approximate timing of 69% of pupping with median pupping date of 26 December [1].

Between 2013 and 2018, HMV *K*. *pneumoniae* infection constituted the majority of pup mortality at Sandy Bay (Fig 4) with a mean annual prevalence of 60.2% (n = 171; Tables 2 and 3). Exclusion of ivermectin-treated dead pups to remove the effects of treatment from the analysis results in a mean prevalence of 62.3%. All cases in this category exhibited gross and histological lesions consistent with HMV *K*. *pneumoniae* infection [7], and cultured a predominant growth of *K*. *pneumoniae* with a positive string test from one or more affected tissues. Overall sex ratio of HMV *K*. *pneumoniae* cases was almost equal at 1.1 (M:F:U 89:81:1), consistent with total necropsied pup deaths of all categories (M:F:U 148:133:3). The first three years of the sample had a male bias (mean M:F 1.66), which in the latter two years reversed to become female biased with a mean of 0.62 (Table 2), but the only statistically significant difference was in 2013–14 (male cases 66% (95% CI 51–79%), female cases 34% (95% CI 21–49%); Table 2).

**Table 3:** Overall cause of death in New Zealand sea lion pups (*Phocarctos hookeri*) necropsied at Enderby Island, including compound diagnoses.

Summarised cause of death	*n*	Cause of death	*n*
HMV *K*. *pneumoniae* infection	171 (60.2%)	HMV *K*. *pneumoniae* infection	139 (48.9%)
		HMV *K*. *pneumoniae* infection and drowning	25 (8.8%)
		HMV *K*. *pneumoniae* infection and trauma	5 (1.8%)
		HMV *K*. *pneumoniae* infection and starvation	1 (0.4%)
		HMV *K*. *pneumoniae* and other infection coinfection	1 (0.4%)
Starvation	42 (14.8%)	Starvation	42 (14.8%)
Trauma/Asphyxiation	28 (9.9%)	Trauma/Asphyxiation	26 (9.2%)
		Drowning	2 (0.7%)
Other infections	20 (7.0%)	Other infections	16 (5.6%)
		Other infections and starvation	3 (1.1%)
		Other infections and trauma	1 (0.4%)
Open	17 (6.0%)	Open	17 (6.0%)
Stillbirth/ Periparturient death	4 (1.4%)*	Periparturient death	3 (1.1%)
		Stillbirth	1 (0.4%)
Congenital	2 (0.7%)*	Congenital	2 (0.7%)

*This value is underestimated as three out of five seasons did not include the dates of peak pupping, therefore stillbirths, periparturient deaths and fatal congenital defects could not have been observed if they occurred.

**Fig 5:**
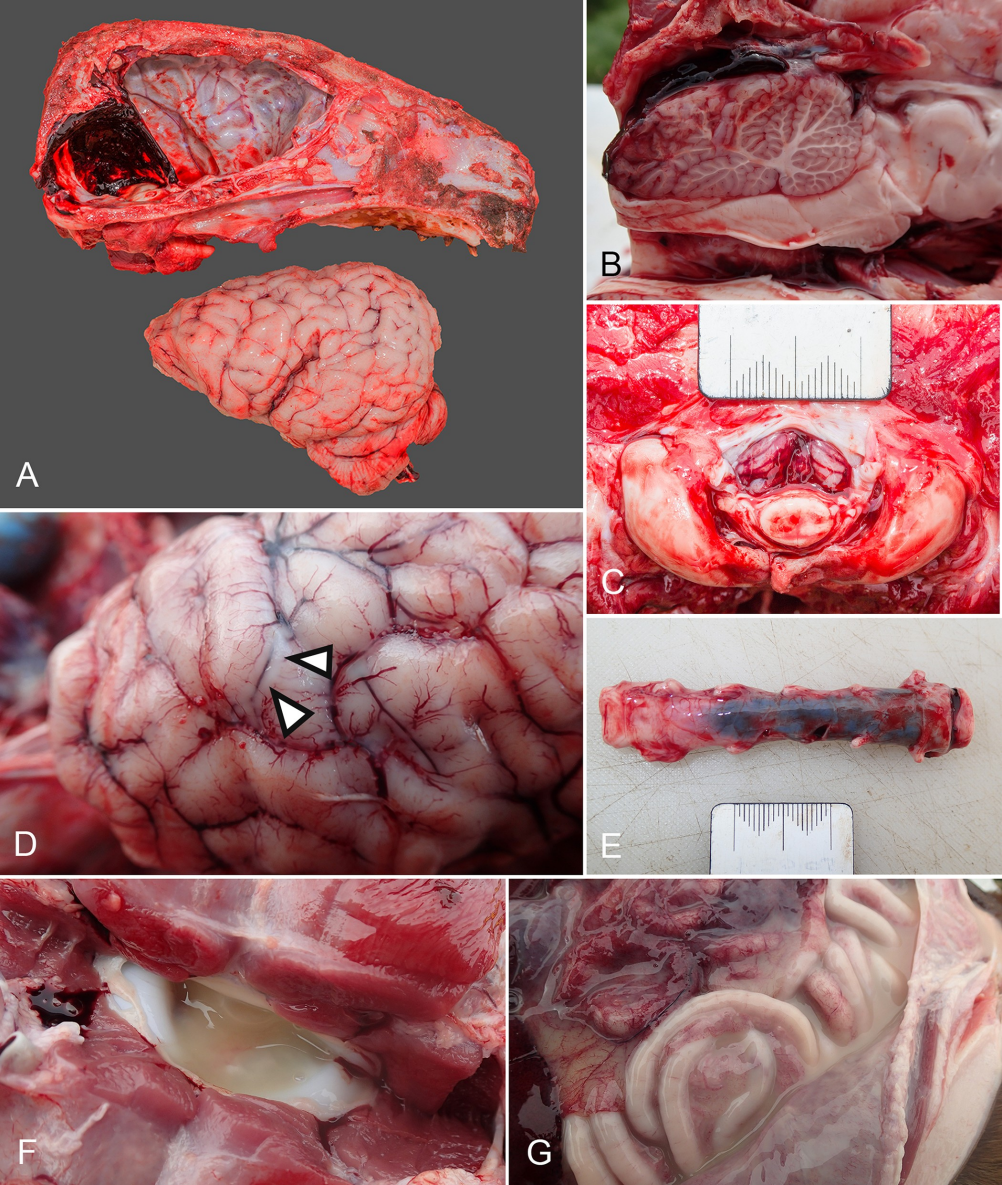
Gross necropsy lesions in New Zealand sea lion pups consistent with hypermucoviscous *K*. *pneumoniae* infection. (A) Subdural haemorrhage, concentrated over the cerebellum. (B) Subdural haemorrhage over the cerebellum in combination with vermis haemorrhage and herniation. (C) Herniation of the cerebellum as viewed from the caudal aspect, on removal of the head at the atlanto-occipital joint. (D) Suppurative subarachnoid plaques, often obscuring underlying vasculature (arrow heads). (E) Cervical spinal cord subdural haemorrhage. (F) Atlanto-occipital septic arthritis with voluminous viscous yellow exudate. (G) Peritoneal effusion and peritonitis with voluminous viscous yellow exudate.

Commonly identified gross necropsy lesions from confirmed HMV *K*. *pneumoniae* cases are summarised in Table 4. Central nervous system lesions and septic arthritis were the most common gross pathological findings. Subdural haemorrhage, particularly localised over the dorsal cerebellum (Fig 5A and 5B), and often extending along the cervical spinal cord (Fig 5E) was found in 63.2% and 57.9% of HMV *K*. *pneumoniae* cases respectively. Subarachnoid suppurative exudate over the cerebrum, settling in sulci, often obscuring vasculature (Fig 5D) and forming thick plaques over the rostral and dorsomedial aspects of the cerebral hemispheres (purulent meningitis) was also found in 52% of HMV *K*. *pneumoniae* cases. Cerebellar herniation was a less common finding (15.2% of HMV *K*. *pneumoniae* cases; Fig 5C). Septic arthritis was identified in 79 *Klebsiella* cases (46.2%), of which 39 cases had a single joint affected, and 40 cases had polyarthritis, the most being seven affected joints in an individual pup. Affected joints demonstrated voluminous viscous yellow exudate (Fig 5F), a common feature of lesions in other sites, including muscle abscesses; pleural, pericardial and peritoneal effusions (Fig 5G) and omphalitis. Single cases of pyelonephritis and oophoritis were found in combination with other lesions. Three cases of hypopyon were identified.

**Table 4:** Summary of common gross necropsy findings in hypermucoviscous (HMV) *K*. *pneumoniae* cases in New Zealand sea lion (*Phocarctos hookeri*) pups at Sandy Bay, Enderby Island; 2013–18.

	Number of pups	2013–14	2014–15	2015–16	2016–17	2017–18
**Total HMV *K*. *pneumoniae* cases**	**171**	**48**	**41**	**13**	**40**	**29**
Purulent meningitis	89 (52.0%)	15	17	8	26	23
Cerebral SDH	42 (24.6%)	13	13	6	8	2
Cerebellar SDH	108 (63.2%)	33	21	9	24	20
Cerebellar herniation	26 (15.2%)	11	4	4	3	4
Cervical spinal SDH	99 (57.9%)	33	16	7	24	18
Any septic arthritis	79 (46.2%)	14	19	8	19	19
Atlanto-occipital	28 (16.4%)	7	4	3	5	9
Shoulder	16 (9.4%)	3	7	1	4	1
Elbow	18 (10.5%)	0*	0*	5	3	10
Carpal	28 (16.4%)	4	4	4	8	8
Hip	5 (2.9%)	0*	0*	2	2	1
Stifle	14 (8.2%)	0*	3*	2	4	5
Tarsal	29 (17.0%)	6	8	4	7	4
Temporomandibular	1 (0.6%)	0	0	0	0	1
Cellulitis/muscle abscess	13 (7.6%)	1	3	0	7	2
Pleural effusion/pleuritis	8 (4.7%)	0	2	0	3	3
Peritoneal effusion/peritonitis	5 (2.9%)	0	1	1	2	1
Pericardial effusion/pericarditis	2 (1.2%)	0	1	0	0	1
Omphalitis	13 (7.6%)	4	1	4	2	2

SDH: subdural haemorrhage.

*Examination of elbow, hip and stifle joints for septic arthritis was inconsistent in 2013–14 and 2014–15.

One case in 2017–18 was diagnosed with a coinfection of HMV *K*. *pneumoniae* and *Streptococcus dysgalactiae equisimilis*. Both isolates were cultured from a stifle joint swab, spleen and brain and both were visualised on Gram stain in brain histopathology sections with minimal inflammation, likely pertaining to the acute course of disease. This case also is the earliest diagnosed HMV *K*. *pneumoniae* case in this five-year series, with the pup found dead on 5 January, almost a month before the expected beginning of HMV *K*. *pneumoniae* cases on the cusp of January and February (Table 2). The 2017–18 season had another isolated ‘early’ HMV *K*. *pneumoniae* case on 11 January, then continued as expected with cases forming the majority of mortality from 1 February onwards (Fig 2).

Almost 15% (25/171) of HMV *K*. *pneumoniae* cases were found dead submerged in pools and streams that should have been escapable and were also diagnosed as having drowned, fulfilling the necropsy criteria of significant aspiration of water or foreign material into small airways. Further discussion of gross and histological findings is elaborated below in ‘Trauma and Asphyxiation’. Conversely, of all HMV *K*. *pneumoniae* cases, 40 (23.4%) were found dead submerged in water but the remaining 15 pups exhibited non-specific findings including stable foam in the respiratory tract without foreign material. These cases could also have in fact drowned but due to the difficulty in confirming this diagnosis, this category should be considered an underestimate.

### Starvation

Approximately 15% (42/284) of pups were classified as having died of starvation. Another four pups were diagnosed with starvation in combination with a systemic infection: HMV *K*. *pneumoniae* septicaemia (n = 1), septic arthritis and cellulitis caused by a coinfection of *Streptococcus phocae* and *Staphylococcus schleiferi* (n = 1) and septic arthritis caused by a bacterial isolate unable to be identified by MALDI-TOF MS (n = 2, see ‘Other Infections’). Localised infection in the latter cases inhibited the pups’ ability to move and nurse, and may have caused the starvation in the latter cases, while histological assessment of the HMV *K*. *pneumoniae* case showed infection to be peracute, so the pup may have died of starvation alone, had it not contracted *K*. *pneumoniae*.

Animals in this group had mean axillary blubber depth of 1.29mm (compared to HMV *K*. *pneumoniae* infections 17.5mm, other infections 6.4mm, trauma/asphyxiation 11.5mm). Pups that died of starvation were often dehydrated with generalised skeletal muscle atrophy, a small thymus, liver and spleen; pyloric melena without any obvious gastric ulceration and absence of gastrointestinal contents. Sixteen cases had been identified up to two weeks before death appearing weak and gaunt, with some attempting to nurse from unrelated females, subadult males or yearlings and in the late season, travelling several kilometres calling for their mother. The latter pups occasionally exhibited superficial ulceration on their palmar weight-bearing surfaces on necropsy.

### Other infections

Infections other than those caused by HMV *K*. *pneumoniae* accounted for 7% of pup mortality at Enderby Island. Excepting three cases, these occurred early in the field season, before the commencement of HMV *K*. *pneumoniae* mortalities. Though not significant, more than twice as many pups in this group were male (n = 14; 70%, 46–88% 95% CI) compared to female (n = 6; 30%, 12–54% 95% CI). Aetiological agents detected as the cause of death included beta-haemolytic *Streptococcus* spp. (n = 6; Table 5; excludes the coinfection case with HMV *K*. *pneumoniae* and *Streptococcus dysgalactiae equisimilis* described above), non-haemolytic *Streptococcus halichoeri* (n = 1), *Escherichia coli* (n = 3), non-HMV *K*. *pneumoniae* (n = 1) and *Salmonella enterica* serotype Kottbus (n = 1). Three grossly identical isolates from three cases could not be speciated by MALDI-TOF MS techniques and one representative isolate underwent whole-genome sequencing. Kraken2 analysis (database: minikraken2_v1_8GB) found 80.22% of sequencing reads could be assigned to the genus *Psychrobacter*. *De novo* whole-genome assembly showed that the genome was 2.86Mb with GC content of 44.75%. Analysing the 16S rDNA determined the most similar sequence in Genbank (accessed 17.5.2019) was the partial 16S rDNA sequence of *Psychrobacter sanguinis* SVG 1 (Genbank Acc. KP325218), with sequence identities over 99% (1456/1460). This isolate was designated *Psychrobacter sanguinis* SM868. The draft genome sequence of SM868 has been deposited at DDBJ/ENA/GenBank under the accession WFKQ00000000. The version described in this paper is version WFKQ01000000. The other two isolates were designated presumptive *Psychrobacter sanguinis* as they were identical on horse blood agar as non-haemolytic small colonies, oxidase positive and on microscopy were short Gram negative rods. Four cases presented histological findings consistent with infection and inflammation, but no predominant growth was determined on culture.

**Table 5:** Summary of infections other than HMV *K*. *pneumoniae*, that were the cause of death in New Zealand sea lion (*Phocarctos hookeri*) pups at Sandy Bay, Enderby Island between 2013 and 2018.

Case number	Pathology	Organism isolated
E13/14-10Ph	Embolic/interstitial pneumonia, vasculitis	*Escherichia coli*
E15/16-23Ph	Endocarditis, hepatitis	*Escherichia coli*
E17/18-13Ph	Interstitial pneumonia, pleuritis, pericholangitis	*Escherichia coli*
E15/16-15Ph	Abscess, pleuritis, peritonitis	*Klebsiella pneumoniae* (non-HMV)
E17/18-17Ph	Septic arthritis, cellulitis, vasculitis	*Psychrobacter sanguinis*
E16/17-9Ph	Septic arthritis*	*Psychrobacter sanguinis* (presumptive)
E16/17-11Ph	Septic arthritis*	*Psychrobacter sanguinis* (presumptive)
E17/18-14Ph	Muscle abscess, cellulitis, septic arthritis, peritonitis	*Salmonella* Kottbus
E16/17-4Ph	Meningitis	*Streptococcus dysgalactiae equisimilis*
E16/17-10Ph	Cellulitis	*Streptococcus dysgalactiae equisimilis*
E17/18-6Ph	Cystitis, urachitis, hepatitis	*Streptococcus dysgalactiae equisimilis*
E16/17-15Ph	Septic polyarthritis, pleuritis, fasciitis	*Streptococcus halichoeri*
E16/17-12Ph	Meningitis	*Streptococcus phocae*
E16/17-14Ph	Cellulitis and fasciitis	*Streptococcus phocae*
E15/16-14Ph	Muscle abscess and septic arthritis*	*Streptococcus phocae* / *Staphylococcus schleiferi*
E15/16-5Ph	Meningitis	No predominant growth
E15/16-16Ph	Pleuritis, mediastinitis, muscle abscess, enteritis	No predominant growth
E16/17-20Ph	Hepatitis	No predominant growth
E17/18-7Ph	Cellulitis and septic arthritis	No predominant growth

*Diagnosis was compound with starvation also a contributing factor to death.

### Trauma and asphyxiation

**Fig 6:**
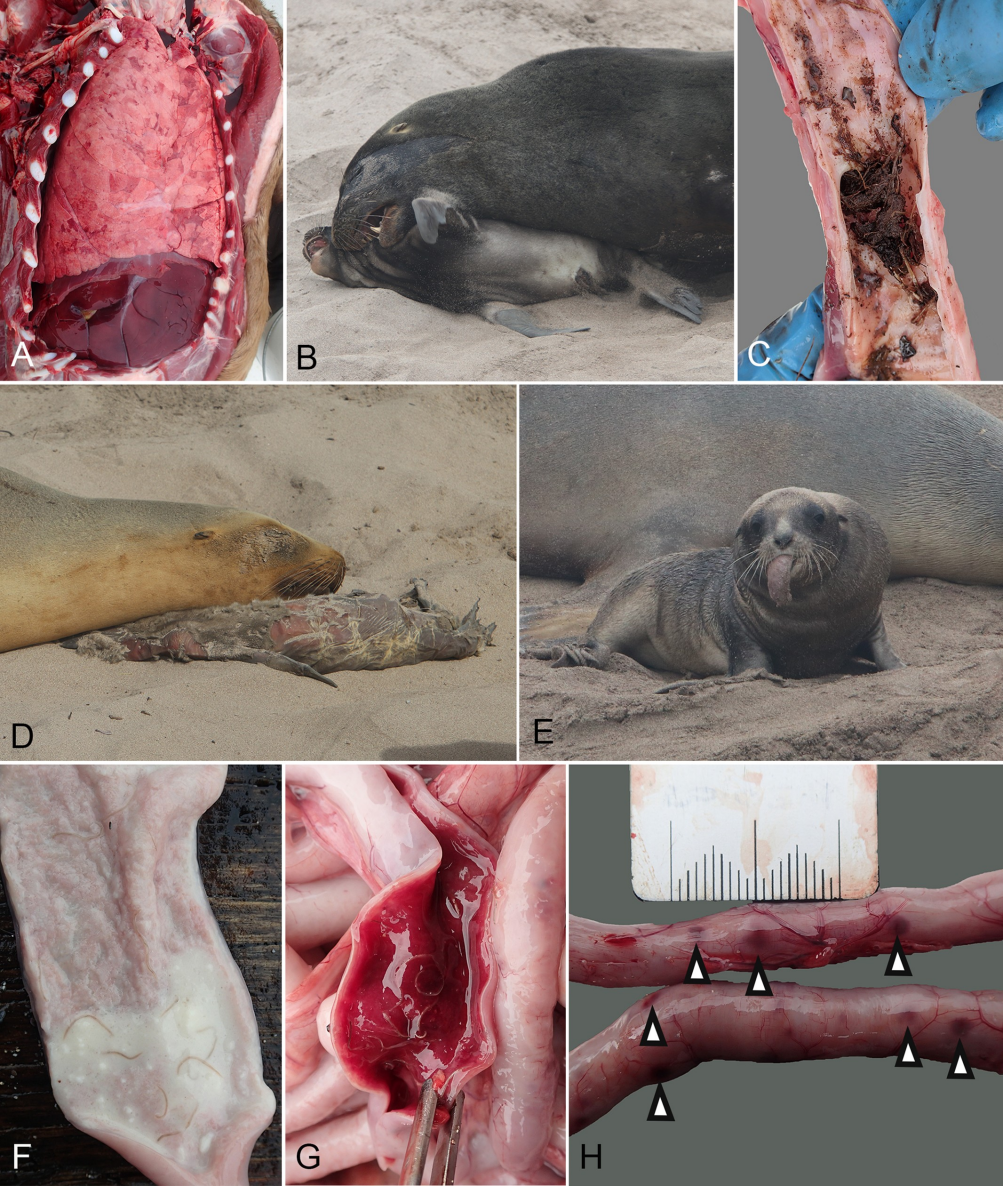
Gross necropsy lesions and clinical presentations in New Zealand sea lion pups consistent with causes of mortality other than hypermucoviscous *K*. *pneumoniae* infection. (A) Hyperinflation of the lungs in a case of drowning, note the lung lobes overlapping and obscuring the heart. (B) A subadult male harasses a pup, a situation that can result in crushing and asphyxiation of the pup. (C) Foreign material including mud and plant material in the trachea, extending into a primary bronchus. This pup was diagnosed with drowning in combination with hypermucoviscous *K*. *pneumoniae* infection. (D) A decomposed stillborn pup that was unable to be retrieved due to the adult female defending the carcass. (E) A pup several days before death exhibiting an elongated tongue and a mass in the left cervical region. The pup was diagnosed at necropsy with congenital rhabdomyoma. (F) Hookworms in the small intestine with milky contents and no haemorrhage. (G) Hookworms in the small intestine with severe haemorrhagic contents. (H) Serosal petechiae on the small intestine (arrow heads).

Twenty eight pups (9.9%) died as a result of trauma or asphyxiation (including drowning). Crushing and blunt trauma causing asphyxiation was overrepresented in the sample with 24 cases compared to two with a sharp aetiology and two drownings. Gross findings in these cases are summarised in Table 6, with drowning cases significantly more likely to exhibit hyperinflation of the lungs (Fig 6A) and aspiration of foreign material including mud, stream water and plant material (*p* <0.001; χ^2^ test; Fig 6C), while blunt trauma cases were significantly more likely to show milk aspiration and bruising to the head and neck (*p* <0.001; χ^2^ test). Sharp trauma cases were infrequent but displayed puncture wounds or muscle damage and haemorrhage consistent with shaking by a subadult or adult sea lion.

**Table 6:** Gross necropsy findings for trauma/asphyxiation cases in New Zealand sea lion (*Phocarctos hookeri*) pups at Sandy Bay, Enderby Island. Pups with a compound diagnosis of hypermucoviscous *K*. *pneumoniae* and drowning are included for completeness.

	Drowning (misadventure)	HMV *K*. *pneumoniae* infection and drowning	All drowning	Blunt trauma	Sharp trauma
*n*	2	25	27	24	2
Hyperinflation of lungs	1	11	12*	1	0
Stable foam in respiratory tract	0	3	3	4	1
Foreign material in respiratory tract	2	23	25*	3	0
Milk in respiratory tract	0	1	1	15*	1
Bruising to head or neck	0	2	2	12*	0
Wound or tissue haemorrhage	0	0	0	2	2

*Significant difference between diagnosis categories *p* <0.001 (χ^2^ test)

Crushing was observed when territorial adult males inadvertently crush poorly mobile neonatal pups in dense harems during breeding activity. Later in the season following dispersal in mid-January, crushing was observed when subadult males attempted mating behaviour on pups (Fig 6B). Both cause death by traumatic asphyxiation, that is, external chest or abdominal compression preventing respiratory movements and venous return to the heart. If the pup had recently nursed, aspiration of stomach contents, usually clotted milk, was commonly identified (n = 15).

Seven pups were found dead in a two-day period during the 2016–17 field season, within an approximately 20m radius of a territorial adult male following dispersal from the beach. This animal was observed mounting and attempting to mate a dead pup (the male estimated >30x heavier than the pup), and consistent necropsy findings of milk aspiration in all seven indicated that death in all occurred by the same means.

Two pups drowned without any evidence of HMV *K*. *pneumoniae* infection. Both cases were found in water bodies with steep undercut sides that unlike the drowned/HMV *K*. *pneumoniae* cases, the pup could not have escaped. Drowning both by misadventure and apparent incapacitation by pain or neurological deficits associated with HMV *K*. *pneumoniae* infection resulted in consistent gross findings in the respiratory tract (Table 6). Hyperinflation of the lungs (Fig 6A) and aspiration of foreign material into the respiratory tract, often with thick mud and plant material obstructing bronchi (Fig 6C) were considered indicative of drowning. Less common findings included subpleural haemorrhage, the formation of a mucus plug at the glottis and stable foam within the respiratory tract.

Two pups were found dead in an identical location two days apart during the 2017–18 field season, having had part of a sandy stream bank collapse on them. Both were diagnosed with asphyxiation with consistent findings of lung hyperinflation, one with extensive petechial subpleural haemorrhages but little debris in the respiratory tract, the other with a marked volume of wet sand filling the trachea and right primary bronchus.

### Stillbirth and periparturient death

Of pups necropsied, one was diagnosed as stillbirth, with consistent necropsy findings as described by Michael and colleagues [14], including uninflated lungs and meconium in the colon. No underlying disease was identified in the sample examined here, however placentas were not collected. Three cases of periparturient death also had meconium in the colon and a non-healed umbilicus, but the lungs floated in formalin (in fresh carcasses), indicating the pup had breathed for a period of time. Six pups that were observed being born dead were not included in further analysis as they were born at the height of the breeding season and could not be safely retrieved for necropsy due to harem density (Fig 6D).

### Congenital

Only two cases of congenital anomalies were identified in the two seasons that researchers were present during pupping. A pup in 2016–17 was observed several times before death exhibiting ataxia and opisthotonus, with a dome-shaped skull. On necropsy, there was bilateral aplasia of the parietal bones of the skull with a remnant midline bony ridge and the brain visible through the dura on reflection of the skin (*cranium bifidum*). There was subdural haemorrhage over the cerebral hemispheres.

A pup in 2017–18 was observed from birth to have a markedly enlarged tongue that could not be retracted into the mouth and severe swelling of the left cervical region (Fig 6E). The pup was able to nurse but died at eight days of age following increasing trauma from sub-Antarctic skua (*Catharacta antarctica lonnbergi*) during maternal foraging trips. On necropsy the tongue had been scavenged and the skull and mandible were malformed with an overall deviation to the right. The neck mass was composed of grossly well organised muscle tissue that on histopathology was consistent with a rhabdomyoma.

### Hookworm infection

Hookworms were present in 66% (132/200) of cases at necropsy where the intestine had not been scavenged. Of these, 65 (49.2%) had haemorrhagic contents, 27 (20.5%) of these with moderate or severe haemorrhage (Fig 6G). Hookworm was not considered to be the primary cause of death in any cases as there were concurrent more severe pathology (usually associated with systemic HMV *K*. *pneumoniae* infection). Of HMV *K*. *pneumoniae* deaths that had intact intestinal tracts at necropsy, 83.7% (103/123) had hookworms present. Of these, 53 cases had intestinal haemorrhage (21 cases of which has moderate or severe haemorrhage). All cases that had intestinal serosal ecchymoses (Fig 6H) in association with hookworm infection (n = 13) died of infectious causes (12 HMV *K*. *pneumoniae* and 1 *E*. *coli*). The median date of the first necropsy case identified with hookworms in the intestinal tract was 13 January (range 12–19 January). No hookworms were identified as having penetrated the intestine to enter the peritoneal cavity on visual inspection, but abdominal lavage was not undertaken. There was a significant correlation (*p* <0.001, χ^2^ test) between hookworm carriage and HMV *K*. *pneumoniae* as the cause of death, but temporally both occur from late January onwards.

## Discussion

This comprehensive suite of diagnostic tests shows infectious aetiologies are responsible for the vast majority of NZ sea lion pup mortality during the study period, with HMV *K*. *pneumoniae* septicaemia and associated foci of localised disease the definitive cause of death in the majority of cases. A much higher proportion of bacterial infection cases (HMV *K*. *pneumoniae* and other infections combined 67.2% compared to 24%) and a lower prevalence of trauma (9.9% compared to 35%) was seen in this study compared to the only comparable previous report [6]. Unlike previously, where hookworm was considered a primary diagnosis in 13% of cases [6], in no cases in this five-year period was the severity of hookworm enteritis considered sufficient to have caused death: instead all had a more severe disease process (usually HMV *K*. *pneumoniae* septicaemia) occurring concurrently. In the absence of routine histopathology and microbiology to investigate tissues beyond those with grossly suppurative lesions at necropsy, early investigations of pup mortality at Enderby Island likely misdiagnosed HMV *K*. *pneumoniae* cases with subdural haemorrhage as mortality due to trauma [5, 6]. Scavenged cases were also excluded from previous analyses, when in fact the body sites more frequently affected by HMV *K*. *pneumoniae* septicaemia: the brain and joints, are rarely able to be accessed by the common scavengers, sub-Antarctic skua and northern giant petrels (*Macronectes halli*), often enabling diagnosis. While the impact of the HMV *K*. *pneumoniae* epizootic event on NZ sea lions was conveyed in these earlier studies, the magnitude of HMV *K*. *pneumoniae* mortality is likely to have been underestimated.

Since the first record of HMV *K*. *pneumoniae* causing mass pup mortality at Enderby Island during the 2001–02 season, this agent has become enzootic; it is now considered the primary cause of pup mortality accounting for almost two thirds of pup deaths at the site annually. HMV *K*. *pneumoniae* mortality has also been recognised at other NZ sea lion breeding sites including Dundas Island, the largest breeding colony for the species (unpublished data) and Otago Peninsula [7], causing comparable lesions in pups; as such it should be considered a serious threat to pup survival in the species. Further, the agent has been detected at Campbell Island in substrate [19] and rectal swabs from adult female New Zealand sea lions [20], but logistical constraints have complicated recent mortality studies at the site and prevented assessment of the role of HMV *K*. *pneumoniae* in pup mortality. Although the initial years of HMV *K*. *pneumoniae* occurrence at Enderby Island in 2001–02 and 2002–03 were deemed ‘epidemics’ with overall pup mortality totalling 31.3% and 22.1% respectively [6], current pup mortality estimates are often well within the range of the second ‘epidemic’ year, being surpassed in three out of five years of this study. The cause of the ‘spike’ of HMV *K*. *pneumoniae* pup deaths during the 2013–14 season is not known and there were not any unusual identified host factors, climatic or environmental variables present. Genomic analysis is ongoing to determine whether there was increased virulence of these *K*. *pneumoniae* isolates. Although field season timing varied, this data shows that overall pup mortality is much higher than pre-epizootic levels and is continuing at levels consistent with the year after the initial discovery of the pathogen in the species. Low juvenile survival (to two years of age) has been shown to be an important contributor to the overall decline of the species ([21], but see [22]), of which pup mortality mediated by HMV *K*. *pneumoniae* is a likely primary factor, and ongoing risk beyond the field season in which cases can be investigated and counted, should be considered.

Temporally, the beginning of the rise in HMV *K*. *pneumoniae* cases each year in this study (with the exception of two outliers in 2017–18) was consistently on the cusp of January and February, later than previous reports of the epizootic season 2001–02 and onwards where *Klebsiella* deaths began on the cusp of December and January [5–7]. This may indicate evolving dynamics of pathogen virulence, environmental load and host carriage factors that should be investigated in future studies. Although pup mortality appears to wane in early March (Fig 4), this is confounded by emigration of pups from locations where researchers can locate and resight them (by mid-March, less than 5% of pups born at Sandy Bay remain there; unpublished data). Pups become increasingly mobile from late January onwards, both unaccompanied and under maternal guidance [23]. They can be found throughout the southern rātā (*Metrosideros umbellata*) forest, with common native shrub species creating dense barriers which pups regularly inhabit but are difficult for researchers to pass without crawling, limiting comprehensive survey of these areas for dead pups. Additionally, pups disperse by sea to adjacent islands in Port Ross (Rose Island, Ewing Island, Ocean Island; Fig 1B; pers. obs), where they cannot be easily accessed to collect survival data. During the dispersal phase, pups are frequently seen with clinical signs consistent with septic arthritis and meningitis (unpublished data), but due to the conclusion of the research season or the dispersal of the animal, a final outcome is unable to be recorded. Based on observations of the clinical course of disease in pups that are able to be located once dead, these symptomatic animals are likely to die as a result of their disease and estimates of pup mortality due to HMV *K*. *pneumoniae* presented here should be considered an underestimate. Consequently, mortality data for the limited window of information available during the summer field seasons provides an important but incomplete insight into the outcomes of a complex annual system of HMV *K*. *pneumoniae* dynamics at this site.

Hypermucoviscous *K*. *pneumoniae* disease has only ever been recorded in the pup age class (< approximately 3 months) of NZ sea lions, despite routine necropsies of adults and juveniles that die at Enderby Island during the field season and on mainland NZ throughout the year [24]. One brief reference to an adult male NZ sea lion found dead at the Catlins, mainland NZ, in 2003 with a pharyngeal abscess described a culture of *K*. *pneumoniae* but the mucoviscosity of the isolate and other necropsy findings were not reported [5]. Although other species may play a role [25], apparently asymptomatic adult NZ sea lions have been shown to harbour HMV *K*. *pneumoniae* in their rectal flora and may provide a route of exposure for pups [20]. The pathogen has been shown to be unable to survive in environmental substrate at temperatures that occur in the NZ sub-Antarctic during winter, suggesting that HMV *K*. *pneumoniae* is introduced to the breeding site by adults as they arrive every December for the breeding and pupping period [19, 20]. Territorial adult males in particular have been shown to travel readily between remote colonies during the pupping period and to the far reaches of the species’ range by the end of breeding in mid-January, potentially exposing pups at all sites [26].

In comparison to other comprehensive pup mortality investigations in otariids around the world, none appear to have such a large infectious disease component as seen in NZ sea lions. In northern fur seals (*Callorhinus ursinus*) on St Paul Island, Alaska; infectious disease accounts for just 3% of cases examined, with emaciation the most common cause of death seen (53%), followed by perinatal mortality (including congenital anomalies and stillbirths; 19%) and trauma (18%) [27]. Improved overlap of the field season with the pupping period at Sandy Bay could demonstrate higher perinatal mortality than reported in this study but based on our observations during full seasons in 2016–17 and 2017–18, this is not a significant cause of mortality for NZ sea lion pups. South American fur seal (*Arctocephalus australis gracilis*) pups at Guafo Island, Chile have been shown to die most commonly with lesions indicative of the hookworm enteritis-bacteraemia (HEB) syndrome (29%), followed by starvation (24%), drowning (21%) and trauma (20%) [28]. Many NZ sea lion pups fulfil the case definition of HEB, being haemorrhagic enteritis in combination with systemic inflammation with bacteria, including periportal hepatitis, interstitial pneumonia, meningitis, encephalitis, vasculitis or peritonitis [29]. Seguel and colleagues [30] undertook microbiological analysis on this species over an extended time period and showed that isolates from HEB cases were predominantly opportunistic commensals including *E*. *coli* and non-HMV *K*. *pneumoniae*. Similar microbiological findings were reported in California sea lions when HEB was first proposed by Spraker and colleagues [31]. It is unclear if there is synergism between hookworms and systemic HMV *K*. *pneumoniae* infection in NZ sea lion pups, but it is interesting that all pups with the most severe hookworm lesions seen, serosal petechiae and ecchymoses, died from systemic bacterial infection: twelve cases of HMV *K*. *pneumoniae* and one case of *E*. *coli* septicaemia. Similarly, an ivermectin treatment trial conducted in the latter two seasons of this study showed a concurrent attenuation of the late season spike in HMV *K*. *pneumoniae* cases (Fig 2). Analysis is ongoing for assessment of risk factors for mortality due to HMV *K*. *pneumoniae*, including hookworm carriage, and results will be presented elsewhere. Pup mortality studies have been conducted in South American fur seals in Uruguay and Australian sea lions (*Neophoca cinerea*) on Kangaroo Island, South Australia but these studies did not utilise histopathology or microbiology so are not comparable here [32, 33].

Drowning in association with HMV *K*. *pneumoniae* infection was a consistent cause of death (9%) between 2013–18. Enderby Island has many undercut streams and sinkholes, likely a result of a combination of peat substrate and regular rainfall [34]. These sites have previously been identified as contributing to pup mortality, accounting for the two uncomplicated drowning cases described in this study, with historic mitigation comprising manual rescue of pups several times daily by researchers (pers. obs). Wooden ramps were installed in pools without natural escape routes throughout the 2014–15 field season, then monitored and refined in seasons following, as a route for pups to self-rescue and have been successful [35]. The cases of drowning with HMV *K*. *pneumoniae* infection described refer to pups found in pools or streams that should be escapable for a healthy pup with normal locomotion. It is hypothesised that pups enter the water to quell pyrexia (>40°C; unpublished data) that develops following HMV *K*. *pneumoniae* infection. Pups then are unable to escape the water due to neurological abnormalities such as ataxia or seizures, weakness, pain or musculoskeletal impairments such as septic polyarthritis or cellulitis. This study has shown that the vast majority of these cases had underlying HMV *K*. *pneumoniae* infection as a contributing factor. Fifteen HMV *K*. *pneumoniae* cases were found submerged in streams or lakes but were not classified as having drowned, as they did not have suggestive necropsy findings such as gross aspiration of foreign material and water into the lower respiratory tract. They are categorised separately to highlight the potential that drowning in combination with HMV *K*. *pneumoniae* infection could be even more prevalent than identified here. The true extent is unclear due to the difficulty of diagnosing drowning on necropsy [36]. An alternative explanation could involve dragging of dead pups into water bodies during the process of scavenging, however this seems unlikely for this number of pups.

Amongst infections caused by bacteria other than HMV *K*. *pneumoniae*, most represented opportunistic commensals or environmental contaminants with beta-haemolytic streptococcal infections the most common. Streptococcal isolates have been reported previously in NZ sea lion pups but were only occasionally classified beyond genus level [6]. Beta-haemolytic streptococci are an emerging cause of morbidity and mortality in marine mammals worldwide, in particular *Streptococcus phocae*, which has been reported to cause bronchopneumonia, septicaemia, abscesses and meningitis in various marine mammal species of north-eastern Canada [37] and septicaemia and abscesses in southern sea otters (*Enhydra lutris nereis*) of California, USA [38]. Both studies demonstrated a correlation with traumatic wounds, which is consistent with the bacterium lacking virulence factors that allow invasion of intact tissue [38, 39]. Cases in NZ sea lion pups did not have any wounds present at necropsy (although may have at the time of infection), but presented with similar gross pathology including cellulitis, abscess formation and meningitis. Neonatal pups are at high risk of invasion by such agents, as well as other opportunistic environmental bacteria as they have a fresh umbilicus in almost continuous contact with sand until it heals up to two weeks later and they regularly mouth other pups and substrate. Taurisano and colleagues [37] further propose that *S*. *phocae* may be a commensal of the female urogenital tract which may provide increased opportunities for pups to become infected. While fresh wounds created by pectoral flipper tagging for permanent identification could provide an entry point for opportunistic pathogens, in the current study, only one of the affected pups had been tagged by the time of death.

Interestingly, non-haemolytic *S*. *halichoeri* was the cause of death in one pup presenting with similar lesions to HMV *K*. *pneumoniae* septicaemia, including septic polyarthritis, pleuritis and fasciitis. *S*. *halichoeri* was first reported as a new species isolated from undefined samples from live and dead grey seals (*Halichoerus grypus*) in the United Kingdom [40]. Since then, the isolate has also been reported in culture from homogenised kidney of a Steller sea lion (*Eumetopias jubatus*) necropsy case in South Korea, but the tissues were too severely autolysed to associate the agent with any pathology [41]. Recent cases published with associated details on pathology associated with infection with *S*. *halichoeri*, in a European badger (*Meles meles*)[42] and several humans [43–45] showed similarities in presentation with the NZ sea lion pup case, often exhibiting suppurative respiratory lesions, sepsis and, in one case, cellulitis. *S*. *halichoeri* was cultured in one other NZ sea lion pup case in this sample, from an omphalitis lesion. While the infection was unrelated to mortality as the cause of death was asphyxiation, it may have become significant with time.

Between 2013 and 2018, there was one case of salmonellosis in a pup, causing severe cellulitis and abscessation of the left hindlimb extending from the pelvis to the tarsus, with concurrent septic peritonitis. Culture and serotyping yielded a diagnosis of *Salmonella* Kottbus, an isolate that has not been reported in marine mammals previously. Asymptomatic intestinal carriage of *Salmonella* is not uncommon in pinnipeds [46–48] and serotypes Cerro, Newport and Derby have previously been isolated from NZ sea lion pup faeces [6].

Three cases of septic arthritis cultured predominant growths, identical in culture, that were unable to be identified by the MALDI-TOF database. Whole-genome sequencing identified the agent as *Psychrobacter sanguinis*, a new species reported in 2012 [49]. *Psychrobacter* spp. are psychrotrophic (cold tolerant) and have been isolated from various marine sources [50]. *P*. *sanguinis* is likely opportunistic and has been described causing cellulitis [50] and nosocomial meningitis [51] in humans. Two of the three NZ sea lion cases had a concurrent diagnosis of starvation and the third case with associated cellulitis was in poor body condition (blubber depth 3mm), indicating that the agent in pups is not highly virulent but more likely to be causing localised infection and debilitation, inhibiting movement and nursing.

Four cases with pathology consistent with infectious processes did not culture any predominant organisms. The collection and storage method for microbiological samples used in this study may not be ideal for some of the less hardy organisms but is necessary given the logistical challenges of the remote field work. It is possible for these cases that the aetiologic agent was or became non-viable and therefore unable to be cultured or was not susceptible to the culture conditions provided. Viruses, fungi and protozoa were not routinely investigated with microbiological techniques but no histological lesions suggestive of these aetiologies were identified. Molecular or genomic techniques would be necessary to further elucidate possible agents involved.

Starvation was the second most prevalent cause of death in this study. Early starvation cases may indicate early abandonment, possibly by inexperienced mothers or a failure of imprinting; later cases could be associated with the wide dispersal of the species on land and failure of the mother to locate the pup which commonly move hundreds of metres while unattended. Alternatively, these mothers could have died either at the colony or away at sea. This is certainly the case for several adult females that are confirmed to have died from *Mycobacterium pinnipedii* infection at Sandy Bay and were known to have had a pup (unpublished data). The pathologic findings in these cases were consistent with those reported for northern fur seal and California sea lion pups diagnosed with starvation and cases of canine starvation by exogenous causes [27, 31, 52].

Whilst trauma accounted for a small proportion of pup deaths compared with findings by Castinel and colleagues [6], a similar temporal trend was noted with initial crushing by territorial males in harems then a second wave of ‘abductions’ by subadult and non-dominant males, although here in mid- to late January rather than late February. In contrast to 20 years ago, the density of the breeding population and therefore harems at Sandy Bay is greatly reduced, perhaps having a protective effect on pups from crushing in the first weeks of life. Few traumatic deaths were seen after late January as the colony becomes dispersed over a wide area of grassland and forest habitats.

In conclusion, these data at Enderby Island show that HMV *K*. *pneumoniae* is now by far the most important threat to pup survival at the site, accounting for over 60% of pup deaths and should be considered a critical target for active mitigation. Work is underway to investigate the risk factors for HMV *K*. *pneumoniae* carriage and mortality in pups and the potential synergistic relationship with hookworm could be amenable to positive manipulation with ivermectin. These results highlight the value of a full investigation of pup mortality including clinical observations, gross necropsy, histopathology and microbiology in determining the true prevalence of causes of mortality in an endangered pinniped population.

## Supporting information

S1 Table List of dead New Zealand sea lion (*Phocarctos hookeri*) pups included in this study, with associated demographic information and cause of death diagnosis (compound and summarised). (XLSX)
